# Conducting a clinical study: A guide for good research practice

**DOI:** 10.4103/0019-5413.30522

**Published:** 2007

**Authors:** Rudolf W Poolman, Beate Hanson, Rene K Marti, Mohit Bhandari

**Affiliations:** Department of Orthopedic Surgery, McMaster University, Hamilton, Ontario, Canada; * Department of AO Clinical Investigation, Davos, Switzerland; ** Department of Academic Medical Center, University of Amsterdam, The Netherlands

The number of prospective randomized trials in orthopedic surgery is increasing.^1^ To assure that the rights, safety and wellbeing of trial subjects (i.e. patients) are protected, the guideline for good clinical practice (GCP) was developed.^2^,^3^ This guideline has its origin in the Declaration of Helsinki. Furthermore, it assures that the clinical trial data are credible. The objective of the International Conference on Harmonization (ICH) GCP guideline is to provide a unified standard for research in Europe, Japan, United States, Australia, Canada and the Nordic countries. A copy of the entire guideline can be downloaded from the following website: http://www.emea.eu.int/pdfs/human/ich/013595en.pdf.

In this article we provide an overview of the guideline for GCP in the context of conducting an orthopedic clinical trial. Our emphasis focuses upon those issues most relevant to orthopedics.

## THE PRINCIPLES OF ICH GCP

The key principle of the GCP guideline is the ethical conduct of a trial.^3^ The trial cannot be initiated before all foreseeable risks and inconveniences are weighted against the anticipated benefit for the individual trial subject and society. Patient safety is the cornerstone of the GCP guideline.^3^ The safety and wellbeing of the trial subjects are most important and should prevail over the interest of science and society.

The available nonclinical and clinical information on an investigational product should be adequate to support the proposed randomized controlled trial (RCT).^3^ Therefore, the developers of the protocol should perform a systematic review (and/or meta-analysis) to gather the available evidence. Orthopedic devices are sometimes introduced into the market without thorough evaluation. This approach may result in unnecessary risk for patients.^4^,^5^ In addition, not all new surgical treatments are improved from current standards.^6^

Investigators considering a new study must ask the key question: do we need a research trial to examine if a treatment works or doesn't? Is there sufficient uncertainty about the relative effects of “investigational” and current standard treatments on patient importance outcomes? To ensure that no relevant published studies already exist, a thorough literature search is mandatory.^7^ Investigators should use a systematic approach to find the available evidence. Often, a systematic review may already exist. The Cochrane library is one extensive database of systematic reviews on a variety of topics.^7^ The conduct of a meta-analysis helps to systematically appraise the available evidence as well as to frame an answerable research question for future research.^8^

Clinical trials should be scientifically sound and described in a clear, detailed protocol.^3^ The British Medical Journal mandates the protocol of a trial to be reviewed together with the final manuscript to identify protocol deviations.^9^ The trial should be conducted in compliance with the protocol that has received prior institutional review board or independent ethics committee approval. Some journals facilitate review of a protocol before the trial starts.^10^,^11^ Protocol publication ensures that investigators follow the guidelines of the protocol they initially proposed. Furthermore, it reduces the potential for sudden changes to the protocol and random analyses (i.e. “data-dredging”) to find a difference between treatment groups.^10^,^12^ Observational studies or case series are most suspect to data-dredging and *post-hoc* revisions.^10^ Therefore, protocols of these studies should be published before the conduct of the study. Legislation in some countries now requires registration of trials. Details can be found at: http://www.controlled-trials.com/.

## METHODOLOGICAL ISSUES IN CLINICAL STUDIES

Key aspects of a methodological sound study design are randomization, allocation concealment and blinding.^13^ Randomization ensures that both known and *unknown* prognostic factors are equally distributed in the treatment and control groups. Allocation concealment prevents undermining of random, unpredictable assignment sequences resulting in overestimated treatment effects.^14^ Blinding of outcome assessors is mandatory, especially if soft outcomes are used, as is often the case in orthopedic trials.^15^ Treatment effects are known to be overestimated in un-blinded studies.^13^,^16^,^17^

### Key administrative issues

The following principles are straightforward but should be followed in every trial. The medical care given to and medical decisions made on behalf of, subjects should always be the responsibility of a qualified physician.^3^ Each individual involved in conducting a trial should be qualified by education, training and experience to perform his or her respective tasks.^3^ Therefore, copies of licenses should be kept in the trial documentation. Before subjects can be enrolled in your study several prestudy documents must be compiled by the study coordinator and placed in the study operations manual. If this is an FDA trial, a form FDA 1572 (statement of investigator form) which summarizes what the FDA requires for an acceptable clinical study must be completed by the principal investigator (PI). An example and instructions for this form can be found at http://www.ctsu.org/FDA_Form_1572_Final.pdf. Formal instructions and forms can be downloaded from http://www.fda.gov/cder/forms/1571-1572-help.html. Other documents that are necessary to have on record before conducting a study to ensure patient confidentiality and legal protection are summarized in Table 1.

**Table 1:** Prestudy documents

Curriculum vitae (CV) - usually required of the PI and all co- or sub-investigators. CVs should be up-to-date, signed and dated to show that they are current. Lab certifications - licenses, certifications and normal laboratory values must be on file*. Signed protocol - This must be signed and dated by the PI. Financial/certification disclosure - Every PI needs to certify/disclose if he/she has a financial interest in the sponsoring company or device being tested*. Institutional review board or ethical committee approval Study budget Letters of agreement with sponsor* Approved informed consent form

* If applicable, PI: Principal investigator

### Expertise-bias: A key consideration when conducting an orthopedic trial

Skepticism about surgical RCTs found its roots in questions about surgeons' learning skills in new surgical techniques. One can ask if all individuals involved in the conduct of a trial can be qualified by education, training and experience to perform his or her respective tasks? Specifically, if surgeons have to perform a new or technically demanding surgical technique in the trial, expertise becomes an issue. Surgeons might be skeptical about a technique they normally do not perform. To overcome this ethical barrier in surgical RCTs the “expertise-based trial” has been promoted by Devereaux and colleagues.^18^ In an expertise-based randomized trial subjects are allocated to a treatment provider, not to a treatment.^18^ This ensures treatment by qualified and motivated surgeons who believe in the technique they are using, thus reducing potential bias.

### Informed consent

Freely given informed consent should be obtained from every subject prior to clinical trial participation.^3^ In orthopedic RCTs patient recruitment can be difficult.^19^–^21^ Low recruitment can lead to small sample size and therefore, insufficient statistical power. The results of an underpowered clinical trial will not be able to provide clinically important answers the trial was designed for, while it risks adverse events of the participants.^22^ Investigators should try to identify potential recruitment problems beforehand and modify their approaches accordingly.^22^ To secure recruitment in surgical trials prerandomization has been suggested and used successfully.^19^,^20^ This randomization design maximizes the physician-patient relationship.^20^ It is important not to undermine the patient's trust in medical science; therefore informed consent and recruitment are important ethical aspects of a trial.^22^

### Data management

All clinical trial information should be recorded, handled and stored in a way that allows its accurate reporting, interpretation and verification.^3^ This principle will ensure proper adjudication and will help data collection. If any adverse event occurs during the trial this needs to be documented for detailed evaluation. Moreover, it will be easier for investigators to collect the results and analyze them. RCTs almost always have some missing data. Inadequate handling of these missing data in the analysis can cause substantial bias in the treatment effect estimates.^23^ One way of preventing this possible source of bias is detailed and accurate documentation. The success and integrity of a trial depends on the data quality and data management.^1^ Data collection in multi-center trials is challenging. Designated coordinating centers use centralized computer data collection systems that can be fax-based or Internet-based.^1^,^24^ Study documents should be created and are summarized in [Table 2]. Each subject who is enrolled in your study must have a folder that contains documents necessary for patient education, informed consent and proper data collection [Table 3]. Each document within the subjects work folder should have the subject unique identifier. For example, 2C-322 may indicate Protocol 2C, site number 3, subject number 22. The confidentiality of records that could identify subjects should be protected, respecting the privacy and confidentiality rules in accordance with the applicable regulatory requirements.^3^ For this reason subjects should be given a unique identification number as described above. Data will be checked in the coordinating centers for missing information, implausible data and inconsistencies at an early stage. Research coordinators from each clinical site should be contacted by phone to ensure that problems are corrected. Failure to resolve problems urgently will violate the GCP guideline and can result in termination of the trial by the authorities as a worst-case scenario.

**Table 2:** Study documents

Document	Description
Investigator brochure (summary of device or method)*	
Delegation of responsibilities form (from PI to others)	
Source documents	The first recording of any observations made or data generated about a study subject during his/her participation.^8^ Source documentation is the foundation of all studies as they confirm the completeness and accuracy of data collection and that the study follows the protocol and is ethically administered. “Facts About Source Documents” can be found at the following FDA website: http://www.fda.gov/cder/present/dia-699/wollen-dia99/
Subject enrolment forms	Forms listing all subjects to aid in the scheduling of subject visits and serves as a checklist to ensure all necessary CRFs are completed for each visit.
Case report forms (CRF)	Documents that provide for a seamless transfer of data from the source documents to the study database. CRFs are preprinted pages that allow the investigator or study coordinator to document data regarding demographics, medication use, prognostic factors and all follow-up outcomes being measured.
Adverse event forms	Forms that allow for the documentation of medical complaints and possible side-effects of any degree that may or may not be attributed to the study procedure.
Authorized signature record	List of names of those individuals who are authorized to complete or make changes to the CRFs.
Site visit log (monitor log)	A form used to record visits by the study monitor to each study site.
Telephone log	Used to record all telephone contacts that pertain to the study.
All correspondence to and from sponsor*	

* If applicable

**Table 3:** Subject folder

Protocol synopsis - It is a good idea to have a simplified version of the protocol that explains in simple and nontechnical terms the process and timeline the patient will need to adhere to during the duration of the study. Medical release of Information for medical records. Informed consent form (two signed copies – one for the subject and one for the research records). Screening sheet that includes inclusion and exclusion criteria for determining eligibility. Pertinent case report forms. Laboratory, radiographic or other records associated with the study. Compensation vouchers if the subjects are getting paid. Letters or communication to or with the subject. Other subject-specific documents as applicable.

Investigational products should be manufactured, handled and stored in accordance with applicable good manufacturing practice. They should be used in accordance with the approved protocol.^3^ In orthopedic trials this issue should be discussed with, for example, the implant manufacturer and documented accordingly.

Systems with procedures that assure the quality of every aspect of the trial should be implemented.^3^ To facilitate this “study operations manual” is created. This manual will be helpful to oversee the day-to-day operations of a study.^25^ It will provide detailed instructions for all study procedures. This manual should include the step-by-step process for enrolling and following patients, entering and managing data and monitoring the process. Copies of all study materials, including study protocol, consent forms, questionnaires, etc, should also be included in the manual. Procedures for maintaining confidentiality and quality assurance and control should be covered.^25^ [Table 4] summarizes the critical ingredients for the study operations manual.^25^

**Table 4:** Critical ingredients for the study operations manual^25^

Element	Description
Study protocol	Read and understand all sections of the protocol and ensure that all investigators have reviewed it, given input and agreed to following the procedures (signature is recommended and sometimes required)
Compile prestudy documents	These are documents that are absolutely necessary to have on record before conducting a study to ensure patient confidentiality and legal protection.
Create study documents	These are documents that you and all study personnel will be using to document the day-to-day study activities including data collection.
Create subject work folder	Each subject will need a folder that includes all documents they must read, understand and sign.

## COMMITTEES AND TRAIL PERSONNEL TO ENSURE ADHERENCE TO THE GCP GUIDELINE

Especially, the complexity of a multi-center trial requires key organizing committees to overlook the conduct of the trial, to assure patient safety and to limit bias in outcome assessment, in other words: adherence to the GCP guideline.^1^

### Steering committee

The steering committee is responsible for the overall design and conduct of a trial.^1^ Although not all trials require safety monitoring by a formal committee external to the trial investigators, all trials need safety monitoring.

### Data safety monitoring board

A data safety monitoring board (DSMB) is usually required for large multi-center trials that evaluate interventions intended to prolong life or reduce risk.^1^ A DSMB consists of healthcare professionals who are completely independent of the investigators.^1^ The members have no financial, scientific or other conflict of interest with the trial. The DSMB members should have relevant clinical expertise, clinical trial methodology experience, bio-statistical expertise and or experience related to medical ethics.^1^ The DSMB reviews data related to the conduct of the study. Recruitment rates, ineligibility, noncompliance, protocol violations and dropouts are aspects reviewed by the DSMB. The DSMB will make recommendations to the steering committee concerning the continuation of the study.^1^

### Adjudication committee

This committee is designated to review important study end-points reported by the trial investigators. This is to determine whether they meet protocol-specified criteria.^1^ To prevent bias, the adjudication committee is blinded to treatment allocation wherever possible.^15^ If blinding is not feasible bias is prevented due to the independent nature of the adjudication committee. Especially for subjective outcome measures such as fracture healing, bias lures, therefore an adjudication committee can ensure the highest scientific quality of a trial.

### Trial personnel

In a large multi-center trial a methods and coordination center controls the daily trial activities, including: centralized randomization, data management and overall coordination.^1^ A multi-center trial with a sample size over 1000 patients will require two data managers, a bio-statistician, two to three research coordinators and a fulltime administrative assistant.^1^ The study coordinators are vital for the success of a trial.

## CONCLUSION

The guideline for GCP was developed to conduct clinical trials on an ethical basis. For investigators it requires a large time commitment but will result in pure data collection while patient safety is maintained. Adherence to this guideline will safeguard patient trust in science.
